# Immune Reconstitution Inflammatory Syndrome Associated Thrombocytopenia in a Patient with Human Immunodeficiency Virus Infection: A Rare Hematological Manifestation

**DOI:** 10.7759/cureus.1369

**Published:** 2017-06-19

**Authors:** Talal Asif, Badar Hasan, Rehman Ukani, Rebecca R Pauly

**Affiliations:** 1 Department of Internal Medicine, University of Missouri Kansas City (UMKC)

**Keywords:** iris associated thrombocytopenia, iris, thrombocytopenia in hiv

## Abstract

Human immunodeficiency virus (HIV) associated thrombocytopenia was commonly encountered in the era prior to the advent of antiretroviral therapy (ART). With the widespread use of ART, its incidence has significantly declined. Immune reconstitution inflammatory syndrome (IRIS) is an immune dysregulation phenomenon that reveals itself clinically as paradoxical deterioration after the commencement of ART in HIV infected patients. It has a wide variety of clinical manifestations. However, hematologic involvement is rare. Here, we present a very rare case of IRIS associated thrombocytopenia. With this case we intend to create mindfulness of the possibility of IRIS being one of the explanations for thrombocytopenia.

## Introduction

With the advent of antiretroviral therapy (ART), we have seen a progressive decline in morbidity and mortality in patients with human immunodeficiency virus (HIV) and acquired immunodeficiency syndrome (AIDS) [1]. One explanation for this improvement is the partial restoration of a patient’s immune function [1]. However, with the initiation of ART, patients may first experience a clinical deterioration. It is theorized that as the patient’s function improves, it tends to mount a systemic inflammatory reaction against both infectious and non-infectious antigens in the body [2]. This phenomenon is termed immune reconstitution inflammatory syndrome (IRIS). IRIS is typically seen in the first three months after commencing ART [3]. It has a wide spectrum of clinical manifestations ranging from infectious to autoimmune, inflammatory, and malignant conditions [1]. Hematologic manifestations seldom have been described.

HIV-associated thrombocytopenia is a common finding in individuals with HIV, seen in up to 30% of patients [4]. Secondary causes of thrombocytopenia in this subset of patients include opportunistic infections, malignancy, impaired hemopoiesis, medication side effects and thrombotic microangiopathy such as thrombotic thrombocytopenic purpura [5]. After excluding secondary causes of thrombocytopenia, the therapy of choice in these cases is initiation or modification of ART [3].

Here, we present a very interesting case of severe thrombocytopenia after the commencement of ART in a patient with previously normal platelet counts. The temporal association was highly suggestive of IRIS. There are only two previously described case reports of IRIS-related thrombocytopenia. In one of these, the patient was already predisposed due to underlying immune thrombocytopenic purpura (ITP). Our case report raises awareness of the possibility of IRIS causing severe thrombocytopenia and the resulting acute blood loss anemia.. We provide a successful treatment approach to guide practicing physicians.

## Case presentation

A 27-year-old female patient with a past medical history of HIV infection and uterine fibroids presented to our infectious diseases clinic for establishment of care. The patient has been diagnosed with HIV infection two years ago but unfortunately was lost to follow-up and was never treated. She had no complaints at this visit and her clinical exam was unremarkable. HIV viral load was obtained that revealed 1,117,895 copies per microliter (mL) with a CD4 count of 230/mL. The patient was started on Genvoya (Gilead Sciences, Inc., CA) (elvitegravir, cobicistat, emtricitabine, and tenofovir alafenamide). The patient was not taking any other medications.

One month later, the patient presented to the emergency department with generalized fatigue, dizziness, and menorrhagia. The patient reported that for the past one week, she had been having heavy menstrual bleeding with large clots and multiple soaked pads. She also noticed easy bruisability and gum bleeding. On initial examination, the patient was very pale. She had a blood pressure of 115/70 mmHg, temperature of 98°F, respiratory rate of 17/minute, and heart rate of 110/minute. Dermatological exam showed diffuse petechiae and purpura all over her body. There was no lymphadenopathy or hepatosplenomegaly. Cardiovascular and respiratory exam were unremarkable.

On initial laboratory evaluation, the patient was found to have a hemoglobin level of 4.3 g/dL from a baseline 11.4 g/dL. Her platelet count was decreased to 4000/cubic millimeter (cmm) from a value of 200,000/cmm just one month prior. Her coagulation profile including protime (PT), international normalized ratio (INR), activated partial thromboplastin time (APTT), white blood cell count, basic metabolic panel, and liver function tests were within normal limits. Peripheral blood smear showed normocytic normochromic anemia and severe thrombocytopenia.

The patient was hospitalized for further management. Genvoya was continued. The patient was emergently transfused two units of platelets and also two units of packed red cells. Platelet count improved slightly to 6000/cmm upon repeat testing with minimal improvement in menorrhagia.

Infectious disease and hematology consults were requested. After a multidisciplinary meeting, it was determined that given the patient's degree of severe thrombocytopenia, inappropriate response to platelet transfusion and rapid decline after initiation of antiretroviral treatment, the diagnosis of IRIS was likely. Further platelet transfusions were withheld and oral dexamethasone at a dose of 40 mg daily was initiated. With this intervention, her vaginal bleeding subsided and platelet counts began to improve. Four days later, on the day of discharge, patient's platelet count had improved to 223,000/cmm. At two-week follow-up, her platelet count had remained stable.

## Discussion

HIV related thrombocytopenia was commonly encountered in the era before widespread use of antiretroviral therapy (ART) [6]. The cause was postulated to be ineffective platelet production, increased splenic sequestration and the presence of co-morbid conditions such as opportunistic infections, malignancy and liver disease [7]. ART has since been the mainstay of treatment for HIV and its related thrombocytopenia [6]. Most of the published data show greatest benefit with the use of zidovudine (AZT) [6]. Other adjunct therapies that have been used with variable success in the past include glucocorticoids, intravenous immune globulins, and splenectomy [8].

IRIS is characterized by clinical deterioration during immune recovery after commencement of ART. Its etiology is hypothesized to be an inflammatory response to intact antigens [5]. Clinical manifestations of IRIS are protean and are connected to the antigenic target involved [1]. IRIS is classically associated with paradoxical worsening of preexistent infectious processes such as mycobacterium tuberculosis, herpes simplex, hepatitis B, and cryptococcus [1]. There is also a known association with a new diagnosis or exacerbation of autoimmune diseases such as Graves’ disease, rheumatoid arthritis, and systemic lupus erythematosus [1]. Conversely, hematological manifestations of IRIS are rare. Thrombocytopenia has only been described in two previous case reports [5-6]. Given the dearth in clinical literature on this subject matter, case reports such as these are essential to guide practicing physicians when encountered with hematological manifestations of IRIS.

The index of suspicion should be particularly high when a temporal association between induction of ART and thrombocytopenia is observed. Since the diagnosis of IRIS-associated thrombocytopenia is clinical, it is imperative to rule out alternate causes of thrombocytopenia. HIV-related thrombocytopenia is anticipated to improve and not worsen with the introduction of ART [6]. Other potential causes such as opportunistic infections, malignancy, medication side effects, hypersplenism (secondary to liver cirrhosis or HIV itself), and thrombotic microangiopathies should be explored.

There are no standard treatment guidelines for the management of IRIS-associated thrombocytopenia. However, on the basis of our clinical experience and previously published case reports, we recommend continuation of ART. Corticosteroids such prednisone at a dose of 1 mg/kg/day or dexamethasone at a dose of 40 mg/day constitute the first line of treatment [9]. We recommend a rapid taper over a course of 10 to 14 days because of the risk of development of opportunistic infections. Other adjuncts to treatment that may be considered include intravenous immune globulin, thrombopoietin receptor agonists (eltrombopag), anti-D immune globulin, rituximab, and rarely splenectomy [10].

In general, the goals of transfusion remain the same as in any other patient with symptomatic thrombocytopenia. We recommend immediate platelet transfusion in all patients with severe bleeding and a platelet count less than 30,000/microliter [5]. 

## Conclusions

Our case report highlights only the third case in literature of IRIS associated thrombocytopenia. We also demonstrate a successful treatment approach for practicing physicians when encountering by such a case. Given the large disease burden of HIV, case reports like these carry immense significance in helping us with timely recognition and treatment to increase favorable outcomes. 
